# Prediction value of ^18^F-FDG PET/CT intratumor metabolic heterogeneity parameters for recurrence after radical surgery of stage II/III colorectal cancer

**DOI:** 10.3389/fonc.2022.945939

**Published:** 2022-09-08

**Authors:** Xin Liu, Yi-Fan Zhang, Qin Shi, Yi Yang, Ben-Hu Yao, Shi-Cun Wang, Guang-Yong Geng

**Affiliations:** ^1^ Department of Nuclear Medicine, the First Affiliated Hospital of USTC, Division of Life Sciences and Medicine, University of Science and Technology of China, Hefei, China; ^2^ Technical and Quality Department, Zhongke Meiling Cryogenics Co., Ltd., Hefei, China; ^3^ Department of General Surgery, The Fourth Affiliated Hospital of Anhui Medical University, Hefei, China

**Keywords:** heterogeneity, colorectal cancer, recurrence, ^18^F-FDG, PET/CT

## Abstract

**Purpose:**

We explored the predictive effect of intratumor metabolic heterogeneity indices extracted from ^18^F-FDG PET/CT on recurrence in stage II/III colorectal cancer after radical surgery.

**Methods:**

A total of 140 stage II/III colorectal cancer patients who received preoperative ^18^F-FDG PET/CT and radical resection were enrolled. ^18^F-FDG traditional parameters including the maximum standardized uptake value (SUVmax), metabolic tumor volume (MTV), and total lesion glycolysis (TLG) under different thresholds; heterogeneity indices including the coefficient of variation with SUV 2.5 as a threshold (CV2.5), CV40%, heterogeneity index-1 (HI-1) calculated by the fixed-threshold method, and HI-2 calculated by the percentage threshold method; and clinicopathological information were collected. We concluded that relationships exist between these data and patients’ disease-free survival (DFS).

**Results:**

Regional lymph node status (*P* < 0.001), nerve invasion (*P* = 0.036), tumor thrombus (*P* = 0.005), and HI-1 (*P* = 0.010) exhibited significant differences between the relapse and non-relapse groups, while SUVmax, MTV2.5, MTV40%, TLG2.5, TLG40%, CV2.5, CV40%, HI-2, and other clinicopathological factors had no differences between the relapse and non-relapse groups. Multivariate analysis demonstrated that HI-1 (HR = 1.02, 1.00–1.04, *P* = 0.038), regional lymph node metastasis (HR = 2.95, 1.37–6.38, *P* = 0.006), and tumor thrombus status (HR = 2.37, 1.13–4.99, *P* = 0.022) were independent factors significantly related to DFS.

**Conclusion:**

HI-1, tumor thrombus status, and regional lymph node status could predict the recurrence of stage II/III colorectal cancer after radical resection and had an advantage over other ^18^F-FDG PET/CT conventional parameters and heterogeneity indices.

## Introduction

Colorectal cancer (CRC) is the third most common cancer globally. A Chinese report indicated that the occurrence and mortality rates of CRC rank third and fifth in all malignancies in China, respectively, with about 0.37 million newly diagnosed patients and nearly 0.20 million deaths (1). Performing radical surgery is the best treatment at present and is very effective. For patients whose tumors cannot be surgically removed, radiotherapy, chemotherapy, immunotherapy, and targeted therapy can be selected on the basis of the actual situation of patients. Despite advances in therapy, the 5-year survival rate after surgery remains below 50% (2).

Since the invasion depth of stage 0/I CRC is limited to the intestinal wall, the risk of recurrence after radical surgery is shallow. Stage IV CRC cannot be completely resected due to distant metastasis, and the prognosis is clinically known to be poor. Compared to the predictable trend of early and late CRC, stage II/III CRC has a relatively deep invasion, some with regional lymph node metastasis, and 20% to 40% of patients have a postoperative recurrence, although radical resection can be performed (3, 4). Therefore, an effective prediction tool, especially a preoperative prediction tool, is needed to predict the prognosis of patients before surgery for stage II/III CRC patients. For those patients with a bad prognosis, the corresponding adjuvant therapy should be performed before or after surgery to reduce the postoperative recurrence rate and improve survival.

^18^F-2-fluoro-2-deoxy-d-glucose (^18^F-FDG) positron emission tomography (PET/CT) is widely applied in tumors as a systemic, non-invasive test. It can assist in tumor diagnosing, staging, reflecting therapeutic efficacy, monitoring recurrence, and predicting prognosis by mirroring the metabolic activity of the tumor tissue. Previous studies have shown that PET traditional metabolic parameters, including standardized uptake value (SUV), metabolic tumor volume (MTV), and total lesion glycolysis (TLG), can predict the prognosis in several tumors, for instance, breast cancer, stomach cancer, and non-small cell lung cancer (5–7). For colorectal cancer, although ^18^F-FDG PET/CT is currently not recommended as a routine investigation for initial staging, it is recommended when distant metastasis is suspected. Related studies have also indicated that traditional metabolic parameters have certain values in colorectal cancer (8–10). In recent years, intratumor metabolic heterogeneity parameters derived from PET/CT, for example, coefficient of variation and MTV-based linear regression slope, have been certified to report the traits of intratumor heterogeneity partly and bring into play in forecasting prognosis in a few entity tumors (11–13). Nevertheless, the predictive effect of ^18^F-FDG PET/CT intratumor metabolic heterogeneity indices on postoperative recurrence of phase II/III resectable CRC has not been researched enough.

This paper retrospectively studied the prognostic worth of intratumor metabolic heterogeneity indices in stage II/III resectable CRC.

## Materials and methods

### Patients

One hundred and forty pathologically proven consecutive CRC patients who received ^18^F-FDG PET/CT at the First Affiliated Hospital of USTC from January 2015 to March 2022 were studied. The inclusion criteria were as follows: 1) colon or rectum cancer was confirmed by pathology as adenocarcinoma or adenocarcinoma with partial mucinous adenocarcinoma; 2) stage II/III was defined in line with the tumor-node-metastasis (TNM) staging system of the American Joint Committee on Cancer (AJCC) (14); 3) all patients received preoperative ^18^F-FDG PET/CT examination and then radical surgery; 4) no therapy was provided before PET/CT test and operation; and 5) FDG metabolism in the tumor tissue was higher than the background. The exclusion criteria were as follows: 1) CRC with other pathologic subtypes such as neuroendocrine tumor, squamous cell carcinoma, etc.; 2) coexistence of other primary malignant tumors; and 3) patients with stage II/III underwent palliative surgery. Approval for this study was obtained from the Medical Ethics Committee of the First Affiliated Hospital of USTC (2022-RE-079).

### ^18^F-FDG PET/CT *a*cquisition

^18^F-FDG PET/CT scans were executed on the Siemens Biography Sensation 16 PET/CT imager (Siemens Medical Systems Group, Knoxville, TN, USA). Before the examination, the patient fasted for over 6 h. Until blood glucose was below 11.1 mmol/L, ^18^F-FDG 0.1–0.2 mCi/kg was administered through intravenous injection. After resting for 40 min, the patient drank 500 ml of water and urine was drained for the whole body scan. The body scan range was from the upper orbital margin to the inferior inguinal margin. Tube voltage 120 kV, tube current 100 mA, 5 mm thickness, 5 mm interval, 0.75 pitch, and matrix size 512 × 512 were the CT scanning parameters. According to the CT scanning field, PET images were carried out in a 3D pattern, generally scanning 6–7 beds, 2.0 min/bed. Image reconstruction using the ordered subset maximum expectation iteration method, PET image attenuation correction using CT scan data, and PET and CT pictures were automatically created in the Wizard Workstation. Finally, the PET, CT, and fusion images of the cross-section, sagittal plane, and coronal plane were obtained.

### Semiquantitative analysis of ^18^F-FDG PET/CT images

**Figure 1:**
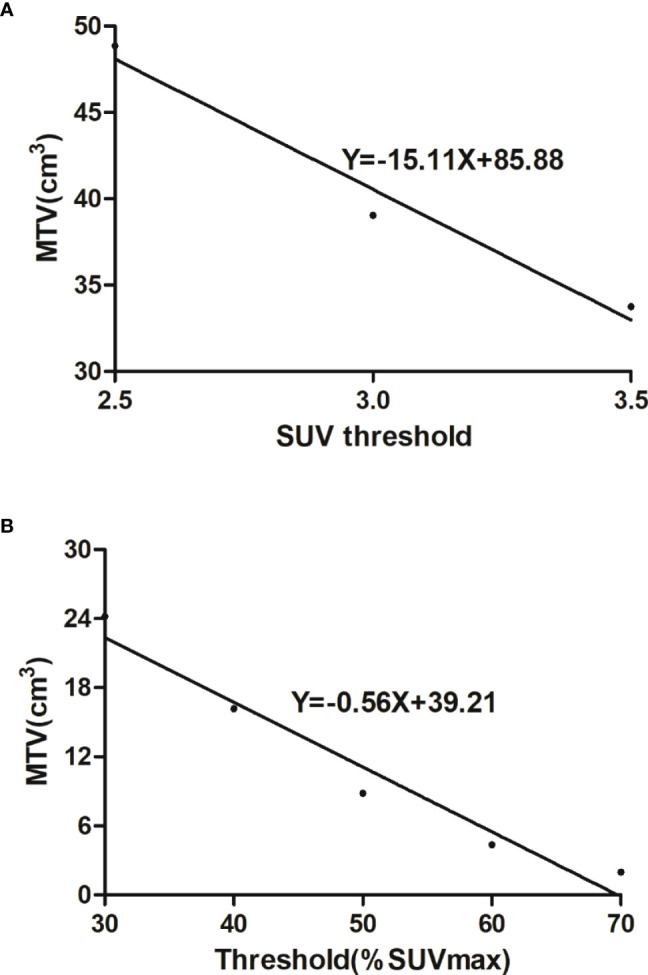
**(A)** Heterogeneity index-1 was the negative form of the linear regression slope calculated by MTVs under three fixed thresholds (2.5, 3.0, 3.5). **(B)** Heterogeneity index-2 was the negative form of the linear regression slope calculated by MTVs under 30%–70% SUVmax thresholds.

The PET/CT images of the primary lesion of colorectal cancer were gauged by the Siemens syngo.*via* workstation (Knoxville, TN, USA). A volume of interest (VOI) at the primary tumor region was set to measure the maximum standardized uptake value (SUVmax). MTV and TLG were counted with 2.5 SUV as a threshold and 40% SUVmax as a threshold, respectively. In addition, we counted the coefficient of variation (CV) and heterogeneity index (HI) as heterogeneity parameters. CV is the ratio of the standard deviation of SUV to SUVmean, which reflects the variation degree of SUV (15). CV2.5 with 2.5 of SUV as the threshold and CV40% with 40% of the SUVmax as the threshold were measured. As the negative form of the linear regression slope of MTV was calculated according to different SUV thresholds, HI represents the MTV discrepancy under different SUV thresholds. Three fixed SUV values (2.5, 3.0, 3.5) were used to create the MTV-based HI-1 (**Figure 1A**) (16), and 30%–70% SUVmax thresholds were used to develop the MTV-based HI-2 (**Figure 1B**) (11). Two well-experienced nuclear medicine experts must be blinded to the patient’s information and separately review the images. In case of disagreement on the images, a high-level doctor would make the final decision.

### Clinicopathologic information

Clinicopathologic information was obtained from the medical records. Clinical data such as gender, age, serum carcinoembryonic antigen (CEA), carbohydrate antigen 19-9 (CA 19-9) levels, and postoperative adjuvant chemotherapy information were collected. Pathological traits were gained from the surgical pathological reports, including the location of the tumor, the largest tumor diameter, pathological types, degree of tumor differentiation, depth of invasion, regional lymph node status (N), nerve invasion status (NI), and tumor thrombus status (TT).

### Follow-up

All patients received radical surgery and achieved a tumor-free state after surgery. Patients were followed up every 3–4 months in the first 3 years and every 4–6 months after that. The follow-up was carried out through clinical interview, serum CEA, CT and MRI examination, and colonoscopy during the review period. Disease progression (recurrence) was affirmed by the Response Evaluation Criteria in Solid Tumors (RECIST) or biopsy pathology of the lesion site. Disease-free survival (DFS) was defined as the time from the radical operation to the first recurrence or death.

### Statistical analyses

Every factor was tested for normal distribution using the Shapiro–Wilk test. The continuous variables with normal distribution were presented as means ± standard deviation (SD), variables that were not normally distributed were presented as medians (interquartile ranges), and categorical variables were presented as proportions. The chi-square test, Student’s *t*-test, and rank-sum test were used to discriminate differences between groups. The relationships between clinicopathologic parameters, PET/CT date, and DFS were evaluated through univariate and multivariate Cox proportional hazards regression. Survival curves were obtained through Kaplan–Meier analysis; the cutoff value for the PET parameters, which most significantly discriminates DFS, was the optimal threshold of each variable; and the optimal threshold of PET parameters was obtained using the receiver operating characteristic curve (ROC). Data analysis was executed using SPSS (version 19.0).

## Results

### Basic information

A total of 140 patients were included in this research. There were 93 (66.4%) men and 47 (33.6%) women, with a median age of 65 years old (range: 34–91). The median follow-up time was 34 months (range: 1–87). Thirty-six patients (25.7%) showed recurrence, and 5 (3.6%) died during the follow-up. Among the recurrent patients, 12 (8.6%) had tumor metastasis to the liver, 10 (7.1%) to the lung, 8 (5.7%) to distant lymph nodes, 3 (2.1%) to the bone, 1 (0.7%) to the spleen, 1 (0.7%) to the abdominal wall, and 1 (0.7%) to the peritoneum. The patients’ basic information is exhibited in **Table 1**.

**Table 1:** Baseline characteristics and PET parameters of the subjects.

Variables	Values
Gender	
– Female	47 (33.6%)
– Male	93 (66.4%)
Age, years	65 (55–75)
Location	
– Right colon	52 (37.1%)
– Left colon	30 (21.4%)
– Rectum	58 (41.4%)
Pathological type	
– Adenocarcinoma	118 (84.3%)
– Adenocarcinoma with partial mucinous adenocarcinoma	22 (15.7%)
Degree of differentiation	
– High	3 (2.1%)
– Medium	95 (67.9%)
– Medium–low	33 (23.6%)
– Low	9 (6.4%)
Measured tumor length (cm)	5.01 ± 1.58
T stage	
– T_2_	14 (10.0%)
– T_3_	84 (60.0%)
– T_4_	42 (30.0%)
Regional lymph nodes	
– N^−^	67 (47.9%)
– N^+^	73 (52.1%)
Nerve invasion	
– Negative	104 (74.3%)
– Positive	36 (25.7%)
Tumor thrombus	
– Negative	96 (68.6%)
– Positive	44 (31.4%)
Stage, AJCC	
– II	60 (42.9%)
– III	80 (57.1%)
CEA (ng/ml)	4.11 (2.47–11.22)
CA 19-9 (U/ml)	15.03 (7.79–30.12)
Postoperative adjuvant chemotherapy	
– No	37 (26.4%)
– Yes	103 (73.6%)
PET parameters	
– SUVmax	16.12 (12.71–22.12)
– MTV2.5 (ml)	44.89 (25.21–66.00)
– MTV40% (ml)	13.13 (8.71–22.49)
– TLG2.5 (g)	248.95 (135.80–433.01)
– TLG40% (g)	134.44 (78.65–220.57)
– CV2.5	0.50 (0.44–0.58)
– CV40%	0.23 (0.21–0.24)
– HI-1	12.26 (7.54–19.36)
– HI-2	0.46 (0.28–0.76)

### Differences of PET/CT indices in different pathologic features

All patients were divided into groups on the basis of different pathologic features. There were no significant statistical differences in all PET indices among different T stages, regional lymph nodes, nerve invasion, tumor thrombus statuses, and different degrees of differentiation. The right colon group had higher MTV2.5, TLG2.5, and TLG40% than the left colon group (*P* = 0.049, *P* = 0.043, and *P* = 0.038, respectively). The adenocarcinoma with partial mucinous adenocarcinoma group had higher MTV2.5, MTV40%, TLG2.5, TLG40%, HI-1, and HI-2 (*P* = 0.004, *P* = 0.002, *P* = 0.016, *P* = 0.010, *P* = 0.006, and *P* = 0.001, respectively) and lower CV40% (*P* = 0.040) than the adenocarcinoma group (details in **Tables 2**, **3**).

Table 2Differences in ^18^F-FDG PET/CT conventional parameters between different pathological features.

**Table:** 

Variables (median)	T_2_	T_3_		T_4_		*P*-value		N^−^	N^+^		*P*-value	TT^−^		TT^+^	*P*-value		NI^−^		NI^+^		*P*-value
SUVmax	13.97	16.60		17.00		0.285		16.75	15.66		0.316	16.33		16.01	0.704		15.59		17.00		0.546
MTV2.5	43.12	41.93		51.13		0.283		44.07	46.55		0.575	46.76		35.57	0.478		46.79		36.39		0.470
MTV40%	15.76	12.79		15.60		0.347		12.30	14.28		0.218	15.11		11.18	0.379		14.86		12.26		0.322
TLG2.5	205.37	235.64		319.78		0.330		243.93	252.81		0.935	274.51		218.28	0.367		278.63		223.76		0.399
TLG40%	122.33	132.54		149.29		0.312		132.65	136.47		0.559	137.29		133.12	0.447		137.79		133.12		0.453

**Table:** 

Variables (median)	Right colon		Left colon		*P*-value		Adenocarcinoma			Adenocarcinoma with partial mucinous adenocarcinoma			*P*-value			HD/MD		M-LD/LD		*P*-value	
SUVmax	17.59		14.91		0.133		15.79			17.10			0.936			16.33		16.01		0.870	
MTV2.5	56.66		42.09		**0.049**		37.95			56.90			**0.004**			45.89		45.35		0.417	
MTV40%	16.02		12.80		0.147		12.34			20.92			**0.002**			12.71		14.53		0.202	
TLG2.5	354.97		201.89		**0.043**		220.10			356.53			**0.016**			248.95		257.36		0.565	
TLG40%	184.85		128.02		**0.038**		129.68			177.64			**0.010**			132.69		140.30		0.334	

HD, high differentiation; MD, medium differentiation; M-LD, medium–low differentiation; LD, low differentiation. Bold value means P <0.05.

Table 3Differences in ^18^F-FDG PET/CT heterogeneous parameters between different pathological features.

**Table:** 

Variables (median)	T_2_	T_3_		T_4_		*P*-value		N^−^		N^+^	*P*-value	TT^−^		TT^+^	*P*-value		NI^−^		NI^+^		*P*-value
CV2.5	0.44	0.52		0.47		0.314		0.51		0.50	0.194	0.49		0.51	0.896		0.49		0.53		0.161
CV40%	0.23	0.23		0.22		0.112		0.23		0.23	0.068	0.23		0.23	0.925		0.23		0.23		0.281
HI-1	16.10	10.94		14.41		0.257		10.56		13.12	0.397	13.00		11.56	0.819		12.71		11.31		0.737
HI-2	0.43	0.42		0.53		0.421		0.40		0.48	0.148	0.48		0.40	0.285		0.48		0.40		0.195

**Table:** 

Variables (median)	Right colon		Left colon		*P*-value		Adenocarcinoma			Adenocarcinoma with partial mucinous adenocarcinoma			*P*-value			HD/MD		M-LD/LD		*P*-value	
CV2.5	0.52		0.49		0.252		0.51		0.50				0.510			0.50		0.51		0.810	
CV40%	0.23		0.23		0.290		0.23		0.22				**0.040**			0.23		0.23		0.931	
HI-1	15.40		11.39		0.114		10.73		17.25				**0.006**			11.35		14.20		0.258	
HI-2	0.56		0.43		0.154		0.41		0.68				**0.001**			0.46		0.47		0.288	

HD, high differentiation; MD, medium differentiation; M-LD, medium–low differentiation; LD, low differentiation. Bold value means P <0.05.

### Clinicopathological parameters, ^18^F-FDG PET/CT indices, and recurrence

Among the clinicopathological parameters, regional lymph node status (*P* < 0.001), nerve invasion status (*P* = 0.036), and tumor thrombus status (*P* = 0.005) differed significantly between the relapse and non-relapse groups. However, gender, age, tumor diameter, CEA, CA 19-9, and other pathological features revealed no significant results.

Only HI-1 (P=0.010) revealed marked differences between relapse and non-relapse groups. All of the metabolic and volumetric parameters and the other heterogeneous parameters including SUVmax, MTV2.5, MTV40%, TLG2.5, TLG40%, CV2.5, CV40%, and HI-2 (*P* = 0.349, *P* = 0.125, *P* = 0.086, *P* = 0.396, *P* = 0.456, *P* = 0.154, *P* = 0.155, and *P* = 0.055, respectively) had no significant differences (**Table 4**).

**Table 4:** Comparison of characteristics and PET parameters according to colorectal cancer recurrence after radical surgery.

Variables	Recurrence (−) (*n* = 104)	Recurrence (+) (*n* = 36)	*P*-value
Gender			
– Male	70	23	
– Female	34	13	0.708
Age (years) (median)	66	61	0.095
Location			
– Right colon	44	8	
– Left colon	22	8	
– Rectum	38	20	0.072
Pathological type			
– Adenocarcinoma	90	28	
– Adenocarcinoma with partial mucinous adenocarcinoma	14	8	0.213
Degree of differentiation			
– High/medium differentiation	77	21	
– Medium–low/low differentiation	27	15	0.076
Measured tumor length (cm) (mean ± SD)	4.98 ± 1.66	5.11 ± 1.32	0.666
T stage			
– T_2_	11	3	
– T_3_	64	20	
– T_4_	29	13	0.638
Regional lymph nodes			
– N^−^	59	8	
– N^+^	45	28	**<0.001**
Nerve invasion			
– Negative	82	22	
– Positive	22	14	**0.036**
Tumor thrombus			
– Negative	78	18	
– Positive	26	18	**0.005**
CEA (ng/ml)	3.98	4.60	0.623
CA 19-9 (U/ml)	14.07	18.49	0.145
Postoperative adjuvant chemotherapy			
– No	28	9	
– Yes	76	27	0.822
SUVmax (median)	16.77	14.93	0.349
MTV2.5 (ml) (median)	44.94	50.56	0.125
MTV40% (ml) (median)	12.96	15.89	0.086
TLG2.5 (g) (median)	244.51	288.38	0.396
TLG40% (g) (median)	136.41	134.44	0.456
CV2.5 (median)	0.51	0.48	0.154
CV40% (median)	0.23	0.22	0.155
HI-1 (median)	11.06	16.59	**0.010**
HI-2 (median)	0.43	0.53	0.055

Bold value means P <0.05.

### Cox proportional hazards regression analysis

**Figure 2:**
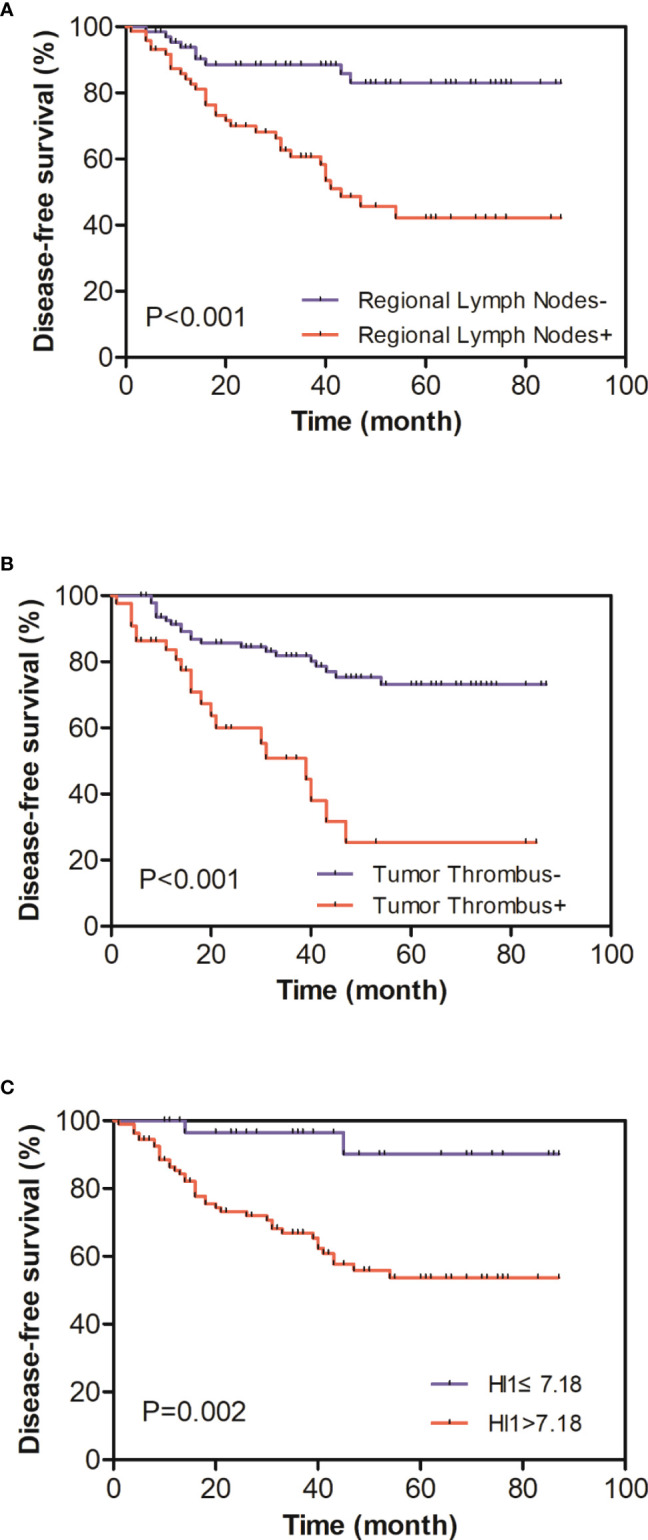
**(A)** Kaplan–Meier curves of disease-free survival (DFS) distinguished by regional lymph node status. **(B)** Kaplan–Meier curves of DFS distinguished by tumor thrombus status. **(C)** Kaplan–Meier curves of DFS distinguished by HI-1 (cutoff 7.18); patients with a higher HI-1 had a more severe prognosis.

Univariate analysis demonstrated that regional lymph node metastasis (HR = 4.01, 1.91–4.83, *P* < 0.001) (**Figure 2A**), nerve invasion status (HR = 3.37, 1.75–6.48, *P* < 0.001), tumor thrombus status (HR = 3.76, 2.00–7.05, *P* < 0.001) (**Figure 2B**), and HI-1 (HR = 1.03, 1.01–1.05, *P* = 0.013) (**Figure 2C**) were significantly related to DFS (**Table 5**). In all the elements which had a significant correlation with DFS in the univariate analysis, regional lymph node metastasis (HR = 2.95, 1.37–6.38, *P* = 0.006), tumor thrombus status (HR = 2.37, 1.13–4.99, *P* = 0.022), and HI-1 (HR = 1.02, 1.00–1.04, *P* = 0.038) were independent factors which were significantly related to DFS after adjusting for these factors in the multivariate analysis (**Table 6**). Patients with regional lymph node metastasis were almost triple as likely to experience recurrence compared with patients without regional lymph node metastasis. Patients with tumor thrombus had more than twice the risk of recurrence than patients with a lower counterpart. Patients with higher HI-1 had nearly 100% risk for recurrence compared to those with lower HI-1, with statistical significance.

**Table 5:** Univariate Cox regression analyses for DFS.

Variables	DFS		
	HR	95% CI	*P*-value
Age			
Gender	1.00	0.97–1.02	0.651
– Female	1.00		
– Male	1.00	0.53–1.89	0.994
Tumor location			
– Right colon	1.00		
– Left colon	1.09	0.45–2.62	0.856
– Rectum	1.67	0.83–3.37	0.149
Pathological types			
– Adenocarcinoma	1.00		
– Adenocarcinoma with partial mucinous adenocarcinoma	1.36	0.65–2.85	0.416
Differentiation			
– HD/MD	1.00		
– M-LD/LD	1.59	0.85–2.99	0.146
Tumor diameter	0.98	0.82–1.18	0.867
T stage			
– T_2_	1.00		
– T_3_	1.39	0.48–4.02	0.548
– T_4_	1.34	0.44–4.09	0.602
Regional lymph nodes			
– N^−^	1.00		
– N^+^	4.01	1.91–4.83	**<0.001**
Nerve invasion			
– Negative	1.00		
– Positive	3.37	1.75–6.48	**<0.001**
Tumor thrombus			
– Negative	1.00		
– Positive	3.76	2.00–7.05	**<0.001**
CEA (ng/ml)			
– Normal (≤5.00)	1.00		
– Increased (>5.00)	1.31	0.70–2.46	0.399
CA 19-9 (U/ml)			
– Normal (≤37.00)	1.00		
– Increased (>37.00)	1.28	0.63–2.62	0.494
Postoperative adjuvant chemotherapy			
– No	1.00		
– Yes	0.94	0.47–1.89	0.871
SUVmax	0.97	0.92–1.01	0.137
MTV2.5 (ml)	1.00	1.00–1.01	0.183
MTV40% (ml)	1.01	0.99–1.02	0.346
TLG2.5 (g)	1.00	0.99–1.00	0.863
TLG40% (g)	1.00	0.99–1.00	0.836
CV2.5	0.11	0.01–1.76	0.119
CV40%	0.01	0.01–119.44	0.196
HI-1	1.03	1.01–1.05	**0.013**
HI-2	1.34	0.87–2.06	0.186

HD, high differentiation; MD, medium differentiation; M-LD, medium–low differentiation; LD, low differentiation. Bold value means P <0.05.

**Table 6:** Multivariate Cox regression analyses for DFS.

Variables	DFS		
	HR	95% CI	*P*-value
Regional lymph nodes			
– N^−^	1.00		
– N^+^	2.95	1.37–6.38	**0.006**
Nerve invasion			
– Negative	1.00		
– Positive	1.68	0.79–3.58	0.182
Tumor thrombus			
– Negative	1.00		
– Positive	2.37	1.13–4.99	**0.022**
HI-1	1.02	1.00–1.04	**0.038**

Bold value means P <0.05.

## Discussion

In the present study, the intratumor metabolic heterogeneity parameter HI-1, calculated by the slope of linear regression of different MTV values under three fixed SUV values (2.5, 3.0, 3.5), was a significant predictor for DFS. Compared with the traditional semiquantitative parameters and other intratumor metabolic heterogeneity parameters of PET/CT, HI-1 had an advantage in predicting II/III resectable colorectal cancer postoperation recurrence. In addition, regional lymph node metastasis and tumor thrombus status were independent predictive factors for DFS. As we know, this is the first research to explore the predictive effect of intratumor metabolic heterogeneity parameters derived from ^18^F-FDG PET/CT on survival outcomes (DFS) for phase II/III resectable CRC patients. Moreover, this is also the first study to explore which heterogeneous parameters calculated by multiple thresholds and methods are the most appropriate for colorectal cancer.

Although radical operation is the most effective therapy, recurrent colorectal cancer following radical surgery is common. The recurrence rate reaches approximately 40%–50% and is most common in the first 2 years (17, 18). The prediction value of postoperative recurrence in patients with CRC by ^18^F-FDG PET/CT conventional indices is still controversial, and the results obtained by different studies are not entirely the same. Nakajo et al. surveyed 38 patients with colorectal cancer and found that SUVmax, SUVmean, MTV2.5, and TLG could not affect DFS in colorectal cancer patients (19). By studying 85 patients with phase II and III rectal cancer, Lee et al. pointed out that MTV and TLG were effective predictors of DFS (20). Bang et al. also reached a similar conclusion (21). Lee et al. applied traditional PET parameters under multiple thresholds, such as SUVmax, MTV2.5, MTV3, MTV30%, MTV40%, TLG2.5, TLG3, TLG30%, and TLG40%, to predict the DFS of colorectal cancer. Finally, only TLG2.5 was a significant factor (10). In our study, SUVmax and the volume parameters, including MTV2.5, MTV40%, TLG2.5, and TLG40%, calculated by the two most commonly used thresholds, were meaningless in discriminating recurrence with statistical significance and were not effective factors for predicting DFS, which was consistent with the results of some literature studies. The different results may be caused by slightly different research objects, sample sizes, and processing methods.

Tumor heterogeneity means that during the tumor growth process, its daughter cells’ features, for instance, genetic expression, cell multiplication, speed of growth, angiogenesis, necrosis, and oxygen deficit, will be changed after multiple proliferation, resulting in discrepancies in invasiveness, drug susceptibleness, and prognosis. Tumor heterogeneity is one of the characteristics of malignant tumors and may correlate with progressive disease, the malignant behavior of cancer, poor response to treatment, and bad prognosis (22).

Therefore, it is possible to evaluate the heterogeneity of tumors by performing the tumors’ heterogeneous metabolism analysis on PET/CT. ^18^F-FDG PET/CT mostly applies traditional parameters including SUVmax, MTV, and TLG to quantify intratumoral heterogeneity. SUV only reflects activity at one point within the tumor. MTV and TLG can report not only the activity information but also the volume information in the entire tumor. However, these parameters cannot discriminate the heterogeneity of different sections in the tumor. CV and HI can solve this problem to some extent. CV reflects the variation of SUV in different areas of cancer, and HI demonstrates the variation of MTV in different cancer regions. Some studies have shown that CV and HI can reflect heterogeneity features such as gene mutation and prognosis in some tumors (16, 23–25). To avoid the different prediction effects in tumors caused by heterogeneous parameters calculated by different thresholds and methods in previous studies, we deliberately chose the two most commonly used thresholds to calculate CV and adopted the commonly used percentage thresholds method and the improved fixed-threshold method to calculate HI.

However, the CV did not get similar prediction results to previous studies. Lee et al. found high CV values related to epithelial ovarian cancer relapse (13). Chung et al. pointed out that CV was the only independent risk factor for early-stage uterine cervical cancer relapse (24). In our research, CV did not differ significantly between recurrence and no recurrence patients and was not the risk factor for II/III resectable colorectal cancer DFS. CV2.5 showed no significant difference among all pathological groups. CV40% was only significantly different between different pathological types of colorectal cancer. Adenocarcinoma with partial mucinous adenocarcinoma, widely known as the higher degree of malignancy, showed lower CV40%. In contrast, as the degree of malignancy was relatively lower, the CV40% of adenocarcinoma was higher (CV40%: 0.23 *vs*. 0.22, *P* = 0.040). This is consistent with Liu et al., who found that the CV of patients with the KRAS mutation group was significantly lower than that of patients with the KRAS wild type (16). CV was not significantly different from other pathological features in this research. The upper results suggest that CV2.5 and CV40% may not be suitable for reflecting intratumor heterogeneity of colorectal cancer.

HI is another type of heterogeneity parameter. HI-1 and HI-2 were counted by using the fixed-threshold method and the percentage thresholds method, respectively. The statistical analysis of HI-1 and HI-2 showed different results. In comparison between groups with various pathological features, HI-1 and HI-2 were significantly different only between different pathological types, indicating that the heterogeneity of adenocarcinoma with mucinous adenocarcinoma was higher than adenocarcinoma. Still, there was no significant difference between HI-1 and HI-2 among other pathological features. In comparing the recurrence and non-recurrence groups, relapse patients had higher HI-1 and HI-2, and the non-relapse patients had lower HI-1 and HI-2, but only HI-1 had a significant difference. In the univariate and multivariate analyses of DFS, only HI-1 was an effective predictor, and HI-2 was not an adequate predictor. The difference between HI-1 and HI-2 may be related to the thresholds selected during calculation. The thresholds chosen for HI-1 are relatively low (2.5–3.5), which can reflect the heterogeneity of the whole tumor, including the lower metabolic site, while the thresholds selected for HI-2 are relatively high (30%–70%), which can only report the heterogeneity of the higher metabolism site inside the lesion. It is known that the distribution of ^18^F-FDG PET activity is highly correlated with glucose metabolism, necrosis, vascularization, and angiogenesis, and glucose metabolism in the necrotic and new parts is relatively low. Therefore, the fixed-threshold method with a lower calculation threshold is more accurate in accurately reflecting the heterogeneity within the whole tumor.

Previous studies have suggested that CEA, N stage, and stage of the tumor are all considered positive predictors of colorectal cancer recurrence (26–28). Our study found that CEA had no significant difference between groups and was not an independent predictor of DFS. At the same time, regional lymph node and vascular tumor thrombus status were different between recurrence and no recurrence groups and were independent predictors of DFS. The CEA level reflects tumor load. If radical surgery removes all lesions, the ability of CEA to reflect tumor burden will be weakened and so will the impact on survival. Regional lymph node statuses determine tumor stages in phase II/III colorectal cancer patients. Patients with regional lymph node metastasis have a higher tumor stage and, therefore, a higher risk of recurrence, which is consistent with previous literature reports. Tumor thrombus is classified into lymphatic and vascular thrombus, which reflects the possibility that the tumor is biologically more invasive and is associated with reduced survival (29). Adjuvant chemotherapy significantly affects survival outcomes in colorectal cancer (30). However, no significant correlation was found between postoperative adjuvant chemotherapy and recurrence in our study, which may be related to the poor compliance of some patients or the incomplete and non-standard course of adjuvant chemotherapy.

This study also had some limitations. Firstly, this research was a single-center, small-sample size study with a short follow-up period, and only DFS could be used as the follow-up outcome. Multi-institutional studies with larger sample sizes and overall survival as the outcome need to be further carried out. Secondly, patients might be treated with various adjuvant therapy regimens after radical surgery. We only considered partial information about whether patients received postoperative adjuvant chemotherapy in the statistical analysis, which could have confounded the prognostication. Thirdly, in this study, HI-1 calculated based on the values of 2.5, 3.0, and 3.5 as the thresholds was a significant risk factor in the final multivariate analysis, but the HR was relatively low. HI, calculated by which thresholds may have a higher HR value and better predictive efficacy, needs to be further explored in the follow-up study. Finally, the partial volume effect can cause distributions of measured intensities to appear more heterogeneous, and we did not perform partial volume correction.

## Conclusion

In general, the ^18^F-FDG PET/CT linear regression HI-1 calculated based on different MTVs under three fixed SUV values (2.5, 3.0, 3.5) was an independent predictor of recurrence for patients with stage II/III resectable colorectal cancer. Preoperative evaluation of HI-1 can well reflect intratumor heterogeneity and, thus, infer the prognosis of cancers because of its characterization. At the same time, regional lymph node status and tumor thrombus status could also predict DFS for phase II/III resectable CRC patients.

## Data availability statement

The raw data supporting the conclusions of this article will be made available by the authors, without undue reservation.

## Ethics statement

This study was reviewed and approved by The research protocol was approved by the Medical Ethics Committee of the First Affiliated Hospital of USTC (2022-RE-079). Written informed consent for participation was not required for this study in accordance with the national legislation and the institutional requirements.

## Author contributions

G-YG and S-CW conceived of the presented idea and performed manuscript editing. XL wrote the original draft. B-HY performed the computations. Y-FZ, QS, and YY collected the data. All authors contributed to the article and approved the submitted version.

## Funding

This study was supported by the National and Provincial Key Specialty Construction Plan (Grant number: Z155080000004).

## Conflict of interest

B-HY is employed by Zhongke Meiling Cryogenics Co., Ltd.

The remaining authors declare that the research was conducted in the absence of any commercial or financial relationships that could be construed as a potential conflict of interest.

## Publisher’s note

All claims expressed in this article are solely those of the authors and do not necessarily represent those of their affiliated organizations, or those of the publisher, the editors and the reviewers. Any product that may be evaluated in this article, or claim that may be made by its manufacturer, is not guaranteed or endorsed by the publisher.
